# Development and Application of a Fluorescence Quantitative RT-PCR Assay for Detection of Group A Rotavirus Among Pigeons in China from 2023 to 2024

**DOI:** 10.3390/v18060640

**Published:** 2026-06-03

**Authors:** Xiaohui Yu, Cuiyao Zhang, Lu Chen, Jingjing Wang, Yufeng Liu, Yuteng Chen, Jinping Li, Guangyu Hou, Shuang Chang, Leilei Sun, Yang Li

**Affiliations:** 1 China Animal Health and Epidemiology Center, Qingdao 266032, China; yuxiaohui@cahec.cn (X.Y.); zhangcuiyao2001@163.com (C.Z.); cl2437982476@163.com (L.C.); wangjingjing@cahec.cn (J.W.); liuyufeng0824@163.com (Y.L.); chenyuteng1998@163.com (Y.C.); lijinping@cahec.cn (J.L.); houguangyu@cahec.cn (G.H.); 2 Key Laboratory of Animal Biosafety Risk Prevention and Control (South China), Ministry of Agriculture and Rural Affairs, Qingdao 266032, China; 3 College of Animal Science and Technology, Shandong Agricultural University, Taian 271018, China; 4 New Hope Liuhe Co., Ltd., Jinjiang Industrial Park, No. 376, Jinshi Road, Chengdu 610063, China

**Keywords:** pigeon group a rotavirus, fluorescence quantitative RT-PCR, epidemiological survey, genetic phylogenetic analysis

## Abstract

Group A rotavirus (RVA), as an important pathogen causing pigeon diarrhea, has caused substantial economic losses for the pigeon breeding industry in recent years in China. To better understand the prevalence and genetic diversity of RVAs among pigeon flocks in China, a fluorescence quantitative RT-PCR assay for detection of RVAs was developed in this study. The fluorescence quantitative RT-PCR assay showed satisfactory specificity, sensitivity, repeatability, and reproducibility, with a limit of detection (LOD) of 1 × 10^2^ copies/μL for RVA. Then, 645 pigeon swab samples collected from live bird markets in 10 provinces of China from 2023 to 2024 were tested for RVA with the fluorescence quantitative RT-PCR assay established in this study to characterize the current epidemiological status of pigeon-origin RVAs (PiRVAs). The results showed that the PiRVA positivity rate detected by the RT-qPCR assay was 12.24% (79/645) among pigeon flocks in China. Guizhou Province had the highest positive rate of PiRVAs (42.86%) among the 10 provinces sampled. The VP7 and VP4 gene of nine representative positive pigeon RVA samples were amplified by specific primers, and the phylogenetic analysis of these samples revealed that all RVAs among pigeons belonged to the G18P[17] genotype, though with certain genetic variations. This study established a useful tool for diagnosing Pigeon RVAs in practice, and provided valuable information to better understand the epidemiology of Pigeon RVAs among pigeon flocks in China.

## 1. Introduction

Group A rotavirus (RVA) belongs to the genus Rotavirus within the family Reoviridae, and is a non-encapsulated, icosahedrally symmetric, double-stranded RNA virus [1]. The RVA genome consists of 11 segmented double-stranded RNA fragments, coding for six structural proteins (VP1-VP4,VP6, VP7) and six non-structural proteins (NSP1-NSP6) [2]. The structural proteins play a decisive role in viral replication, antigenic properties, and viral genotyping, and the nonstructural proteins main influence virulence and replication processes [3,4,5,6]. Rotaviruses are classified into nine well-defined serotypes, ranging from A to I. Among them, Group A rotavirus is the predominant epidemic strain infecting pigeon flocks [7]. The VP7 protein and the VP4 protein are independent neutralizing antigens that respectively determine the G and P genotypes of rotavirus. Based on the binary classification system of the outermost VP7 and VP4 layers of RVA, 42 G genotypes and 58 P genotypes have been identified to date [8,9]. At present, the pigeon RVAs that have been identified in NCBI include: the P0-13 strain (G18-P[17]-I4-R4-C4-M4-A4-N4-T4-E4-H4), VIC strain (G18-P[17]-I4-R4-C4-M4-A4-N4-T4-E19-H4), DR-5 strain (G18-P[17]-I4-R4-C4-M4-A4-N4-T4-E11-H4), VIC strain (G18-P[17]-I4-R4-C4-M4-A4-N4-T4-E19-H4), GK-684 strain (G18-P[17]-I4-R4-C4-M4-A4-N4-T4-E19-H4), NIE13-A-1026 strain (G18-P[17]-I4-Rx-Cx-Mx-Ax-Nx-Tx-Ex-H19), etc., all of which belong to the G18P[17] genotype [10,11].

RVA is a common pathogen in pigeon flocks, and young pigeons often develop diarrhea, dehydration, and other clinical signs following infection. In 1981, RVA was first detected in the feces of Belgian pigeon flocks. Since then, and foreign scholars have successively conducted research on pigeon-origin RVAs. In 1988, Japanese scholars Minamoto et al. [12] found that the positive rate of RVA in feral pigeons was 32.4%, and successfully isolated two strains of RVA from feral pigeon feces for the first time. Rubbenstroth et al. [13] investigated the infection of RVA in domestic pigeons in some regions of Europe from 2010–2018, and found that the positive rate of RVA in pigeon flocks was 51.56% in Germany, 57.14% in Belgium, and as high as 100% in Denmark. Research on avian rotavirus in China mainly focus on the infection situation of chickens, as well as the molecular biological characteristics of the virus, etc. However, there are relatively fewer studies on rotavirus in pigeons [14].

RVA detection mainly relies on laboratories, and few studies have been conducted on pigeon RVA detection methods. Chinese scientists have reported the detection of pigeon RVA using high-throughput sequencing technology, but this method is complex and expensive. To meet the demand for pigeon RVA detection in China, in this study, based on the progress research on RVA in pigeon flocks at home and abroad, we designed specific primers and probes according to the conserved region of the VP6 gene, and established a fluorescence quantitative RT-PCR assay for RVA in pigeon flocks. This method demonstrated high specificity, sensitivity, and good reproducibility, and has been proven to be applicable for the clinical detection of RVA in pigeons. Using this method, we conducted an epidemiological survey of RVA on 645 oropharyngeal and cloacal swabs of pigeon samples from 10 provinces in China in 2023–2024 to understand the prevalence and distribution of RVA in China. At the same time, gene sequencing and phylogenetic analysis were performed on representative samples of pigeon RVA to improve the gene information database of pigeon RVA in China, and to reveal the molecular evolutionary characteristics of pigeon RVA in China, with the aim of providing molecular epidemiological data for the prevention and control of the disease.

## 2. Materials and Methods

### 2.1. Ethics Statement

This study was conducted in accordance with animal welfare guidelines of the World Organization for Animal Health and approved by the Animal Welfare Committee of the China Animal Health and Epidemiology Center.

### 2.2. Viruses, Primers, and Probes

DNA samples of pigeon circovirus (PiCV), pigeon adenovirus (PiAdV-A and PiAdV-B), and pigeon herpesvirus (PiHV), as well as RNA samples of pigeon rotavirus A (PiRVA), avian influenza virus (H9-AIV), pigeon coronavirus (PiCoV), Newcastle disease virus (NDV), and chicken rotavirus (CRV) were stored at −20 °C in the Avian Disease Surveillance Laboratory at the China Animal Health and Epidemiology Center. For PiRVA, at least 20 genome sequences were downloaded from NCBI for comparative analysis. Primer Premier 5 software was used to design the primers and probes based on their most conserved regions. The PiRVA probe was labeled with CY5 at the 5′end, and with BHQ-1 as the 3′ terminal quencher (Table 1). Primers and probes were synthesized by Sangon Biotech Corporation (Shanghai, China).

### 2.3. Samples Collection and DNA Extraction

Intestinal tissue samples were collected from 40 pigeons with suspected PiRVA infection based on clinical signs including diarrhea, originating from 10 pigeon farms in Shandong Province. Each sample was homogenized in a 1:3 ratio of tissue to phosphate-buffered saline (PBS) containing antibiotics, with a total volume of 1.5 mL per sample. After grinding, the samples were centrifuged at 4 °C and 12,000 rpm for 5 min to obtain the supernatants.

A total of 645 oropharyngeal and cloacal combined swabs (oropharyngeal swab and cloacal swab of one pigeon mixed in one tube) were randomly collected from pigeons without overt clinical signs at live bird markets (LBMs) across 10 provinces in China under a national surveillance program conducted from 2023 to 2024. The swabs were homogenized in 1.5 mL of PBS supplemented with antibiotics and then centrifuged at 4 °C for 5 min at 12,000 rpm. Supernatants from the swab samples were used for RNA/DNA extraction using the FinePure Virus DNA/RNA Column Extraction Kit (JIFAN BIOTECH, Beijing, China).

### 2.4. Development and Optimization of the Fluorescence Quantitative RT-PCR

The concentrations of primers and probes were optimized. After optimization, the 20 μL real-time PCR reaction mixture consisted of the following components: 2 × One step RT-qPCR Buffer II (probe) 10 μL, cRNA templates 2 μL, primers (PiRVA-F/R) (10 μM) 1.0 μL, probes (PiRVA-P) (10 μM) 0.4 μL, Evo M-MLV RTase Enzyme Mix 0.4 μL, Pro Taq HS DNA polymerase 0.4 μL, and ddH_2_O 4.8 μL. The fluorescence quantitative RT-PCR amplification was performed on the Applied Biosystems QuantStudio 5 (Thermo Fisher Scientific, Waltham, MA, USA); the amplification condition was set at 42 °C for 5 min, 95 °C for 20 s, followed by 40 cycles of 95 °C for 5 s and 60 °C for 35 s, and the fluorescent signal was detected at the end of the extension step in each cycle.

### 2.5. Generation of Standard Curves

Amplified fragments with PiRVA F/R were synthesized and cloned into the pEASY-T5 vector. Plasmids were extracted from positive bacterial clones using the TIANprep Mini Plasmid Kit (Tiangen Biotech, Tianjin, China). Positive recombinant plasmids were subjected to single-enzyme digestion with Xba I. The plasmid after enzymatic identification was purified and recovered using the DNA Recovery Kit, and its concentration was determined by NanoDrop Nucleic Acid Quantifier (Thermo Fisher Scientific, Waltham, MA, USA). An appropriate amount of fully linearized recombinant plasmid was used as a template for in vitro transcription using the RNA Production System-T7 kit. The product of the in vitro transcription was then used as a standard positive control. The concentration of the standard product was converted to copy number using the following formula: y(copies/μL) = (6.02 × 10^23^) × (ng/µL × 10^−9^)/(DNA length × 340). The 10-fold serially diluted standard product was used as templates to generate the standard curve of the fluorescence quantitative RT-PCR assay.

### 2.6. Specificity and Sensitivity

To evaluate the specificity of the established fluorescence quantitative RT-PCR assay, RNA samples of PiRVA, AIV, NDV, and PiCoV and DNA samples of PiHV, PiCV, and PiAdV were applied. The sensitivity and limit of detection (LOD) of the fluorescence quantitative RT-PCR assay developed were verified in triplicate by using 10-fold serially diluted standard product.

### 2.7. Repeatability and Reproducibility

To evaluate the repeatability and reproducibility of the fluorescence quantitative RT-PCR assay, five 10-fold serial dilutions of the standard product were used to assess intra-assay and inter-assay variability. For intra-assay variability, the assay was repeated three times for each dilution on the same day. As for inter-assay variability, each dilution was tested in two independent experiments by two operators on different days. Coefficients of variation (CVs) for the Ct values were calculated from both intra-assay and inter-assay results.

### 2.8. Comparison of Fluorescence Quantitative RT-PCR with the Conventional RT-PCR

The fluorescence quantitative RT-PCR assay established in this study was used to detect 40 pigeon clinical samples. The results were compared and analyzed with those of the conventional RT-PCR [15] (Table 2) method and sequencing results commonly used in the clinic.

### 2.9. Prevalence Survey of PiRVA in China from 2023 to 2024

The fluorescence quantitative RT-PCR method established in this study was used to detect 645 swab samples collected randomly at LBMs in 10 provinces of China from 2023 to 2024 with Evo M-MLV One Step RT-qPCR Kit II (Probe). The distribution and positive rate of PiRVA were analyzed.

### 2.10. Sequencing and Phylogenetic Analysis

Nine PiRVA strains isolated from different provinces were selected for sequencing and phylogenetic analysis. The VP4 and VP7 genes of PiRVA were amplified using conventional RT-PCR (Table 3), and the PCR products were analyzed by 1% agarose gel electrophoresis. The amplified products were then sequenced by Tsingke Sequencing Ultra of Qingdao Tsingke Biotechnology Co., Ltd. (Qingdao, China). Sequence alignments and phylogenetic trees were performed by MEGA6.0 software [16], and the neighbor-joining method was employed to construct the genomic evolutionary tree with 1000 bootstrap replicates for validation.

## 3. Results

### 3.1. Standard Curve of the Fluorescence Quantitative RT-PCR

**Figure 1:**
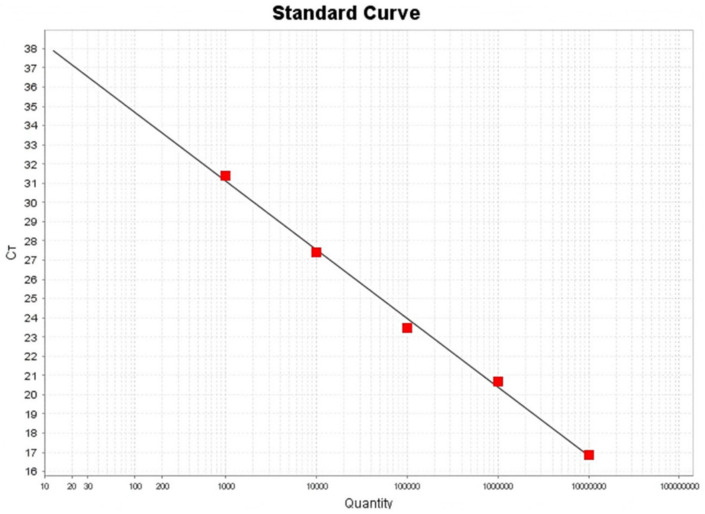
PiRVA the fluorescence quantitative RT-PCR detection standard curve. Standard curve for product Y = −3.571X + 41.815, R^2^ = 0.996.

As shown in Figure 1, the standard curves of fluorescence quantitative RT-PCR assay were generated by using 10-fold serially diluted standard product of PiRVA. The standard curve for a fluorescence quantitative RT-PCR showed that the slope was −3.571, the correlation coefficient R^2^ is 0.996 (Figure 1). The results indicated the high efficiency of the newly developed fluorescence quantitative RT-PCR assays.

### 3.2. Specificity and Sensitivity of the Fluorescence Quantitative RT-PCR

**Figure 2:**
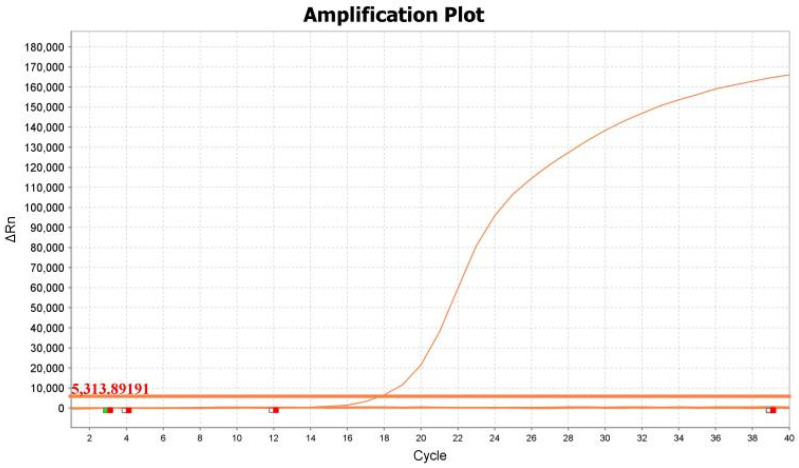
Specificity test of the fluorescence quantitative RT-PCR assay. The fluorescence signals of CY5 were detected by the fluorescence quantitative RT-PCR in PiRVA. No specific fluorescent signal was obtained when other pathogens were tested, including DNA samples of PiCV, PiAdV, and PiHV, and RNA samples of AIV, NDV, PiCoV, and CRV.

The 10-fold serially diluted standard product of PiRVA was used as a positive control, and ddH_2_O was used as a negative control. DNA/RNA samples of other common AIV, NDV, PiCoV, PiCV, PiAdV, PiHV, and CRV were used to assess assay specificity. The amplification curves showed that only the corresponding CY5 signal for PiRVA could be specifically detected, while no fluorescence signal was detected for other viruses (Figure 2). The above results indicated that the established assay had high specificity.

**Figure 3:**
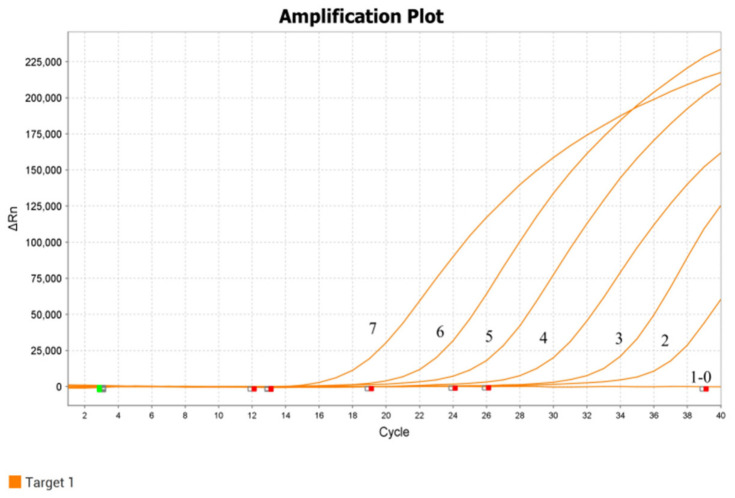
Sensitivity test of the fluorescence quantitative RT-PCR assay. Labels 0–7 indicated the different concentrations of product (10^0^–10^7^ copies/μL).

The sensitivity of the fluorescence quantitative RT-PCR assay was determined by using the 10-fold serially diluted standard template (1 × 10^0^–1 × 10^7^ copies/μL). The ddH_2_O was used as a negative control. Results showed that the LOD of the fluorescence quantitative RT-PCR assay for detecting PiRVA, was 1 × 10^2^ copies/μL (Figure 3).

### 3.3. Repeatability and Reproducibility of the Fluorescence Quantitative RT-PCR

The repeatability and reproducibility of the fluorescence quantitative RT-PCR assay were evaluated by testing different concentrations of the corresponding standard template. The intra- and inter-assay coefficients of variation (CVs) for Ct values for the fluorescence quantitative RT-PCR assay ranged from 0.02% to 0.04% and 0.00% to 0.02%, respectively (Table 4). The results indicated satisfactory repeatability and reproducibility for the fluorescence quantitative RT-PCR.

### 3.4. Comparison of the Fluorescence Quantitative RT-PCR with the Conventional RT-PCR

The fluorescence quantitative RT-PCR method developed for PiRVA in this study and the conventional RT-PCR were used to detect 40 clinically suspected PiRVA infection samples from 10 pigeon farms. The results showed that 15 samples were positive for PiRVA by the fluorescence quantitative RT-PCR method, which was confirmed by sequencing as PiRVA gene fragments. In comparison, conventional RT-PCR identified 12 PiRVA-positive samples (Table 5). The concordance rate between the two methods was 92.5%, with a Kappa coefficient of 0.83. Additionally, the positive samples of these PiRVA infections were obtained from seven pigeon farms. The positive rate of PiRVA infection at the sites of these pigeon farms reached 70% (7/10).

### 3.5. Prevalence Survey of PiRVA in China

**Figure 4:**
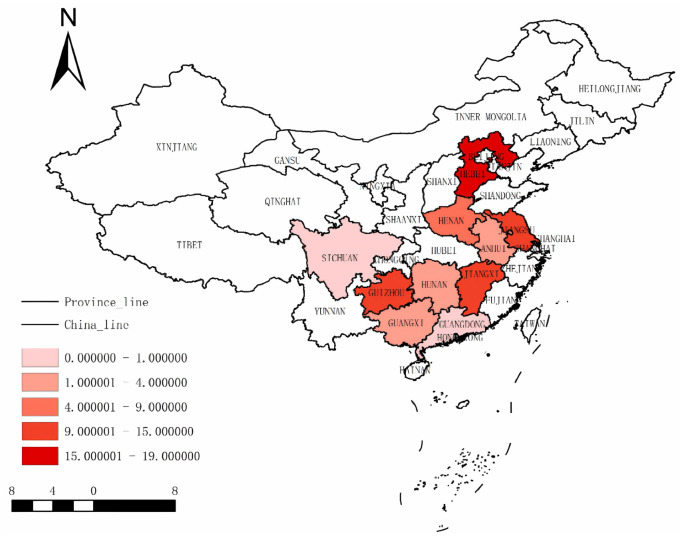
Distribution of PiRVA in 10 provinces of China.

Oropharyngeal and cloacal swabs (*n* = 645) were collected from symptomless pigeons at LBMs in 10 provinces of China from 2023 to 2024. A total of 79 PiRVA positive samples were detected by the real-time PCR assay at 54 different sampling sites in 10 surveyed provinces: Hunan, Henan, Jiangsu, Sichuan, Anhui, Guangxi, Guizhou, Hubei, Guangdong, and Jiangxi. The overall positive rate of PiRVA was 12.24% (79/645) (Table 6). The positive rate was the highest (42.86%) in Guizhou, while no cases were detected in Guangdong (Figure 4). In addition, the 79 RVA-positive samples were collected from 30 sampling sites, including 22 live poultry retail markets and eight live poultry wholesale markets. The site-level statistics showed that the RVA-positive rate of pigeon in live poultry retail markets was 55% (22/40), and this rate in live poultry wholesale markets was 57.14% (8/14), respectively.

### 3.6. Gene Sequencing and Phylogenetic Analysis

**Figure 5:**
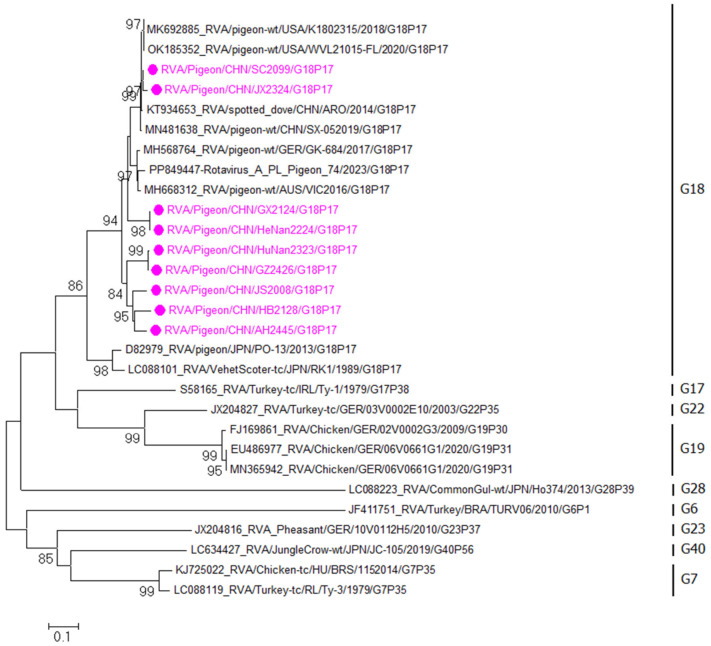
Phylogenetic tree based on the nucleotide sequence of the PiRVA VP7 gene. Samples indicated by “●” represent the positive samples tested in this study.

Nine representative samples from different regions were selected and sequenced, and the sequences were then uploaded to GenBank (Table 7). The nucleotide sequence alignment and the phylogenetic tree of the VP7 and VP4 gene fragments were analysed. Sequences of representative RVA VP7 and VP4 genes published by GenBank were selected and downloaded to construct phylogenetic trees with the VP7 and VP4 genes. The results showed that all nine sample fragments of the VP7 gene collected in this study were classified in the G18 genetic evolutionary branch (Figure 5), and had a relatively distant genetic relationship with other genotype strains. The nucleotide homology of the VP7 gene in the nine samples ranged from 88.3% to 100%, and all were highly homologous to the G18 genotype strains. Among them, the nucleotide homology of the JX2324 strain and the Australian K1802318 strain was 98.3%. The nucleotide homology between the SC2099 strain and the Chinese SX-05 strain was relatively high, at 99.1%. The nucleotide homology between the GX2124 and Henan2224 strains and the German GK-684 strain was 93.5%, and the nucleotide homology between Hunan2224, GZ2426, JS2008, HB2128, and AH2445 strains and the Chinese ARO strain ranged from 90.4% to 91.3%.

**Figure 6:**
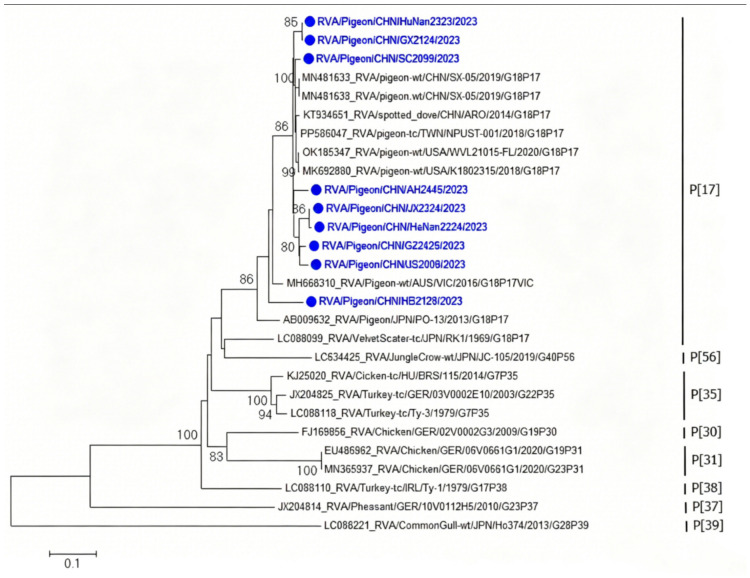
Phylogenetic tree based on the nucleotide sequence of the PiRVA VP4 gene. Samples indicated by “●” represent the positive samples tested in this study.

The nucleotide homology among the VP4 gene fragments of the nine samples ranged from 89.9% to 99.2%, and genetic evolutionary analyses showed that these strains were located in the P[17] genetic evolutionary branch (Figure 6). The SC2099 strain has the highest homology with the NPUST-001 strain from Taiwan, reaching 98.4%, and the GX2124 and Hunan 2323 strains have a homology of 96.4% with the K1802315 strain from the United States. The AH2445 and GZ2426 strains have a homology of 96.2% with the SX-05 strain from China. The JS2008 and JX2324 strains have homologies of 97.1% and 94.8%, respectively, with the ARO strain from China, and the HB2128 strain has a homology of 90.1% with the VIC strain from Australia.

## 4. Discussion

In recent years, with the industrialization and scaling-up of China’s pigeon breeding industry, the degree of automation has been continuously improving, and the number of pigeons bred has been increasing year by year. People are becoming increasingly concerned about whether there are any related infectious diseases among the pigeon flocks. Rotavirus is a common pathogen in pigeon flocks, and sick pigeons mainly exhibit clinical symptoms such as dehydration, diarrhea, and slow weight gain [17]. When pigeons are infected only with RVA, the mortality rate is relatively low. However, when pigeons are infected with RVA and are also attacked by other pathogens, their mortality rate will significantly increase, especially for young pigeons. This has caused serious economic losses to the pigeon industry.

Early detection of the pathogen is crucial for controlling the large-scale infection and spread of RVA in pigeon flocks. There are several clinical methods based on the detection of avian RVA. Among them, electron microscopy is the most effective way to visualize the presence of viral particles in cells, while this method involves a rather cumbersome sample processing procedure. Immunofluorescence and RT-PCR are currently widely used detection techniques. However, both of these techniques require high viral loads for detection, thus their sensitivity is relatively low. Therefore, it is urgently necessary to establish a rapid, sensitive, specific and accurate detection method for detecting RVA in pigeon flocks, in order to provide technical support for the early detection of pigeon origin RVA.

In this study, specific primers and a probe were designed targeting the conserved VP6 gene of PiRVA, and the reaction system and reaction conditions were optimized to establish the fluorescence quantitative RT-PCR method for detection of PiRVA. This method has strong specificity, high sensitivity and good repeatability. Only PiRVA nucleic acid produced a specific amplification curve, and the lowest detection limits were 10^2^ copies/µL. The coefficient of variation of PiRVA was less than 0.04%, which showed good repeatability. The fluorescence quantitative RT-PCR method established in this study and the conventional RT-PCR method were used to detect the etiology of 40 clinically samples suspected PiRVA infection. The results showed that 15 PiRVA positive samples were detected by the fluorescence quantitative RT-PCR method. Meanwhile, all positive samples were confirmed by sequencing. However, only 12 positive samples of PiRVA were detected by conventional RT-PCR. It is suggested that the fluorescence quantitative RT-PCR method for PiRVA established in this study has higher sensitivity and better clinical diagnostic performance than the conventional RT-PCR method. In conclusion, this study established a rapid, sensitive, specific, and accurate fluorescence quantitative RT-PCR detection method for PiRVA, providing necessary technical support for the early diagnosis and comprehensive prevention and control of PiRVA.

In order to understand the prevalence of RVA in pigeon flocks in China, a total of 645 oropharyngeal and cloacal swabs were collected from LBMs in 10 provinces of China from 2023 to 2024, and the fluorescence quantitative RT-PCR established in this study was used to detecting the PiRVA. The result showed that 79 positive samples were detected with a positive rate of 12.24%. The prevalence of RVA in Chinese pigeon flocks was similar to that in Australia, where the individual positive rate of RVA in pigeon flocks reached 13.73% between 2016 and 2017, and in Germany, where the individual positive rate of RVA in pigeon flocks was 10.3% between 2017 and 2018 [18,19]. In this prevalence survey of PiRVA in China, PiRVA were detected in the nine surveyed provinces, indicating a risk of scattered outbreaks. The positive rates of PiRVA varied from 5% to 42.86% across different province, with no positive cases detected in Guangdong Province. Comprehensive prevention and control measures such as strengthening surveillance are needed to reduce the risk of the spread of the disease. In terms of site distribution, PiRVA positivity at pigeon farms was 70% among the 10 farms submitted for RVA testing. In 2018, at pigeon breeding farms in Belgium, the positive rate of the RVA was as high as 57.14%. During the period from 2010 to 2018, the positive rate of RVA in pigeon farms in Germany was 51.56% [13]. The difference in the positive rate of the pigeon farms may mainly be due to the fact that the samples submitted for testing by pigeon farms were all from pigeons suspected of having RVA infection, which resulted in a higher positive rate of pigeon farms in China. In this prevalence survey, PiRVA positivity rates were high in wholesale markets (57.14%) and retail markets (55%), which play an important role in the spread of PiRVA. This phenomenon may be caused by a combination of factors such as the numerous sources of poultry in LBMs, as well as the inadequate implementation of cleaning and disinfection measures. These factors may have greatly facilitated the spread and mixed infection of the virus. Therefore, it is necessary to strengthen market supervision and implement regular market closures.

Phylogenetic analysis and nucleotide homology analysis revealed that all nine representative PiRVA samples in this study belonged to genotype G18P[17]. This is consistent with the relevant conclusions that have been obtained for pigeon RVA worldwide, which all exhibit the G18P[17] genotype [10,20]. However, there are significant differences among these nine samples in terms of homology and genetic relationship. For instance, the VP7 gene of strain SC2099 is closely related to the WVL21015-FL strain from the American pigeon population, while its VP4 gene is more closely related to the SX-05 strain from the Chinese pigeon population. It is speculated that this might be due to the frequent contact and mixed grouping of poultry from different regions and sources, which ultimately led to the occurrence of RVA recombination. Furthermore, studies have shown that when different hosts acquire genomic fragments of RVA from other hosts and can use these fragments as a genetic backbone, it will lead to the spread of this genotype of RVA virus among different species [21,22].

In summary, an RT-qPCR assay for PiRVA detection was successfully developed in this study, which showed good specificity, sensitivity, repeatability, and feasibility. In addition, the prevalence of PiRVA infection in pigeon flocks in China was first evaluated using this established method, and the results showed that PiRVA was widely distributed and has a high positive rate in pigeon flocks in China. The G18P[17] genotype of RVA is the most prevalent genotype among global pigeon populations. Moreover, the RVA virus may undergo genetic recombination and potentially spread across species. Therefore, sustained surveillance and PiRVA vaccine development should be implemented to control PiRVA infection in pigeons in China.

## Figures and Tables

**Table 1:** Primers and probes used in this study. F, R, and P indicate forward primer, reverse primer, and probe, respectively.

Primers/Probes	Sequences (5′–3′)	Genes	Amplicons
PiRVA-F1	GACCAGTRTTTCCAGCWGGTA	VP6	
PiRVA-R1	TCTTGCRTGGGTAGCGCAGAT		202 bp
PiRVA-P1	TTYACAGCAGCATCCATTCGGRGCAT		

**Table 2:** Primers used in this study. F and R indicate forward primer and reverse primer, respectively.

Primers	Sequences (5′–3′)	Genes	Amplicons
PiRVA-F2	GCAGCACCATTTCCWAATCAT	VP6	396 bp
PiRVA-R2	GGTCACATCCTCTCACTATA		

**Table 3:** Primers used in this study. F and R indicate forward primer and reverse primer, respectively.

Primers	Sequences (5′–3′)	Primer Position/nt	Amplicons
VP4-1F	GGCTATAAAATGGCTTCTCTC	1~21	1316 bp
VP4-1R	GTTATYTCRAAGAACGGYTC	1334~1353	
VP4-2F	ATTTAAACCTGCARTTGGC	1315~1333	1071 bp
VP4-2R	GTCACATCCTCATAGACAYCT	2382~2402	
VP7-F	GGCATTTAAAACAGTAATTTTCGT	1~24	1065 bp
VP7-R	GGTCACATCAATCCTTCACAT	1044~1065	

**Table 4:** Intra-repeatability and inter-repeatability of the fluorescence quantitative RT-PCR assay.

Virus	Standard Sample (Copies/μL)	Intra-Repeatability		Inter-Repeatability	
		X ± SD	CV/%	X ± SD	CV/%
PiRVA	1 × 10^8^	12.13 ± 0.21	0.02	13.84 ± 0.14	0.01
	1 × 10^7^	15.18 ± 0.33	0.02	16.95 ± 0.06	0.00
	1 × 10^6^	18.85 ± 0.48	0.03	19.44 ± 0.43	0.02
	1 × 10^5^	21.92 ± 0.46	0.02	22.60 ± 0.28	0.01
	1 × 10^4^	24.97 ± 0.89	0.04	25.28 ± 0.24	0.01

Footnote: X = mean value; SD = standard deviation; CV = coefficient of variation.

**Table 5:** PiRVA the real-time fluorescence quantitative RT-PCR vs. conventional RT-PCR assay comparison of results.

	Positive Sample (Rate)	
	RT-qPCR	RT-PCR
Positive sample (rate)	15 (37.5%)	12 (30%)

**Table 6:** Positive annual rates of PiRVA detection in 2023–2024.

Guangxi	40	2	5.00
Hubei	197	19	9.64
Jiangxi	75	12	16
Anhui	48	3	6.25
Hunan	70	4	5.71
Guizhou	35	15	42.86
Jiangsu	79	14	17.72
Sichuan	15	1	6.67
Guangdong	18	0	0
Henan	68	9	13.23
Total	645	79	12.24

**Table 7:** NCBI accession numbers of PiRVA nucleotide sequences.

	Sequence Accession Numbers (VP4)	Sequence Accession Numbers (VP7)	Location
RVA/Pigeon/CHN/JX2324/2023	PZ407969	PZ441369	JIANGXI
RVA/Pigeon/CHN/GX2124/2023	PZ407970	PZ441361	GUANGXI
RVA/Pigeon/CHN/HB2128/2023	PZ407971	PZ441362	HEIBEI
RVA/Pigeon/CHN/HeNan2224/2023	PZ407972	PZ441368	HENAN
RVA/Pigeon/CHN/HuNan2323/2023	PZ407973	PZ441366	HUNAN
RVA/Pigeon/CHN/AH2445/2023	PZ407974	PZ441363	ANHUI
RVA/Pigeon/CHN/GZ2426/2023	PZ407975	PZ441367	GUANGZHOU
RVA/Pigeon/CHN/JS2008/2023	PZ407976	PZ441365	JIANGSU
RVA/Pigeon/CHN/SC2099/2023	PZ407977	PZ441364	SICHUAN

## Data Availability

The dataset supporting the conclusions of this article is included within the article.
